# A curious case of generalized pruritus in giant cell arteritis

**DOI:** 10.1016/j.jdcr.2026.06.054

**Published:** 2026-06-30

**Authors:** Tylar Dickson, Ndidi Enwereji, Meena Moossavi

**Affiliations:** a Michigan State University College of Human Medicine Flint Campus, Flint, Michigan; b Department of Dermatology, Wayne State University School of Medicine, Detroit, Michigan

**Keywords:** giant cell arteritis, pruritus, tocilizumab

## Introduction

Giant cell arteritis (GCA) is a granulomatous vasculitis that primarily affects medium and large arteries. Etiologies include autoimmune dysfunction, infectious and environmental triggers, and genetic factors.^1^ Common initial features include scalp tenderness, new-onset headache, jaw claudication, fever, fatigue, and weight loss.^2^ Although ocular complications are well-described in GCA, cutaneous manifestations are less documented and range in presentation. Herein, we describe an atypical GCA presentation that spans years of extensive workup prior to diagnosis. This case demonstrates that when managing seemingly disjointed symptoms, reassessment of the leading diagnosis is essential to ensuring further congruous treatment.

## Case report

An 83-year-old African American male presented to the dermatology clinic in May 2023 with a 2-year history of persistent itching. The itching was constant and generalized, but most intense on his back. He reported use of scented body wash and standard laundry detergent and denied daily emollient use. Medical history was notable for rosacea, type 2 diabetes mellitus, asthma, peripheral artery disease, hypertension, and gout. He denied any history of cancer, liver or renal dysfunction, and was negative for other systemic symptoms, such as fever and chills. On exam, no visible or palpable rash was observed. He was diagnosed with generalized pruritus and prescribed twice daily triamcinolone 0.1% ointment as needed, hydroxyzine as needed, and counseled on gentle skin care. Despite this, his pruritus persisted for 2 years.

He also had a complex ocular history followed by ophthalmology, including bilateral open-angle glaucoma managed with twice daily dorzolamide/timolol, bilateral cataracts, and right vitreomacular traction. There was no evidence of diabetic retinopathy.

He was evaluated by rheumatology in May 2025 for bilateral shoulder and hip pain, and a 30-lb unintentional weight loss over the preceding year. Physical exam showed limited shoulder range of motion and reduced hip internal rotation. Laboratory evaluation was notable for elevated erythrocyte sedimentation rate and C-reactive protein, negative rheumatoid factor, and normal creatinine. Polymyalgia rheumatica (PMR) syndrome was diagnosed, which warranted further investigation into possible GCA. Temporal arteritis duplex ultrasonography demonstrated abnormal intimal thickening and bilateral halo signs involving the right parietal and frontal arteries and the left temporal arteries, confirming GCA. He was started immediately on prednisone 40 mg daily following imaging confirmation. Within 1 month, he reported marked improvement in systemic symptoms, including resolution of pruritus.

Intravenous tocilizumab was initiated in July 2025 at 6 mg/kg every 4 weeks. Prednisone was continued until August 2025, at which time it was tapered after 3 weeks of tocilizumab therapy.

Two years following his initial dermatologic visit and following treatment of GCA, he returned to dermatology with complete resolution of pruritus. All dermatologic medications were discontinued, and follow-up was arranged as needed.

## Discussion

This clinical picture incorporates features that are not conventionally associated with GCA. In general, cutaneous manifestations of GCA are rare. Reported dermatologic signs include scalp, tongue, and lip necrosis, cutaneous nodules, and associated diseases such as granuloma annulare and basal cell carcinoma.^3^ These are not inherently pruritic and would not explain our patient’s generalized pruritus. We conducted a targeted literature search to ascertain any reports of GCA-related pruritus. Although this yielded no documented reports, other atypical GCA manifestations, such as a solitary nodule and scattered erythematous-violaceous plaques, were reported.^4^^,^^5^

In contrast, our patient’s pruritus was not associated with a visible cutaneous component. Pruritus is multi-factorial and subjective, both of which make it difficult to delineate a definite etiology and manage in its entirety. Diabetes mellitus, a comorbidity in our patient, is a plausible contributor to his pruritus. However, at his initial presentation in 2023, his diabetes was controlled with empagliflozin and diet. This was reflected in his HbA1C of 5.2, and 5.6 6 months prior to that. Additional laboratory studies from 2023 were unavailable. Early 2025 labs showed high-normal blood urea nitrogen/creatinine and unremarkable hepatobiliary studies, while December 2025 workup demonstrated stage 3b CKD and hepatobiliary abnormalities. Although these findings support pruritic etiologies, their onset after initial symptoms began, along with sustained pruritic relief on tocilizumab, makes these causes less likely.

**Fig 1:**
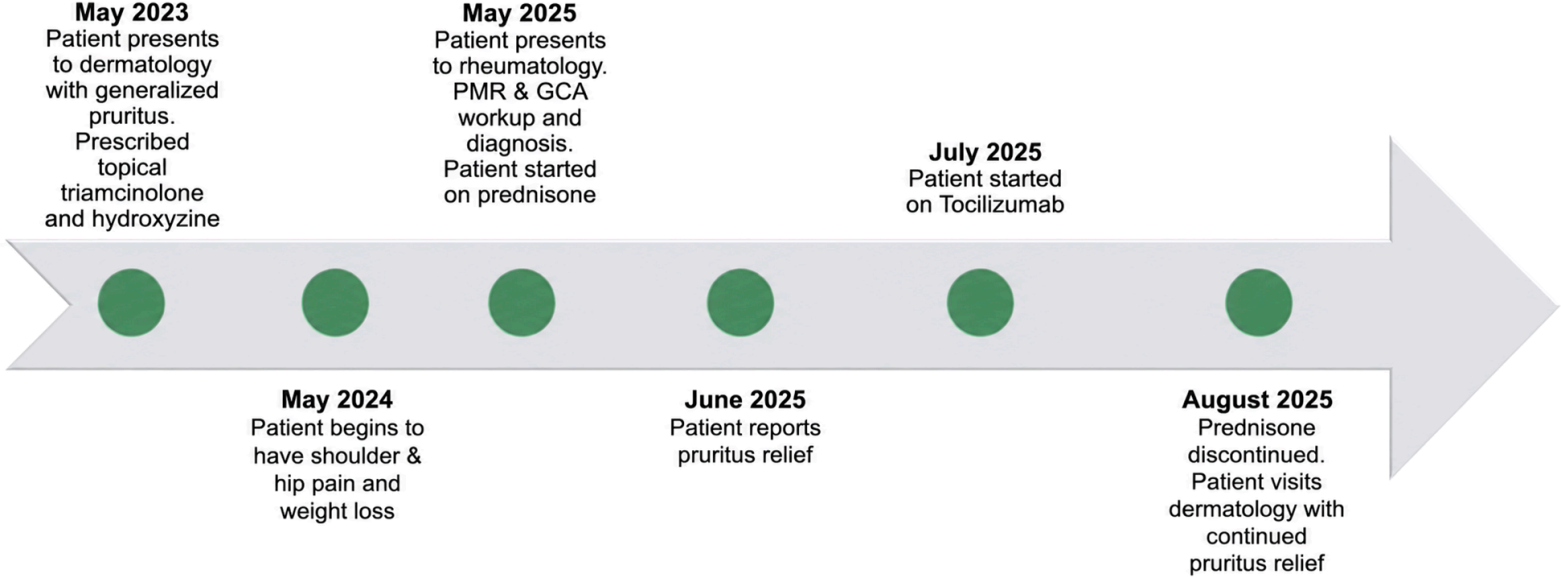
Timeline of patient’s summarized clinical course, including symptom onset, diagnostic evaluation, therapeutic interventions, and subsequent outcomes. *GCA*, Giant cell arteritis; *PMR*, polymyalgia rheumatica.

While no causal relationship between GCA and generalized pruritus has been established, pruritus has been observed in other autoimmune conditions, such as systemic sclerosis and lupus erythematous.^6^ Similarly, no direct link has been established between pruritus and interleukin-6 (IL-6). IL-6 is a prominent proinflammatory cytokine implicated in many autoimmune conditions and is the target of first-line GCA therapy, tocilizumab. In prurigo nodularis and atopic dermatitis, elevated levels of IL-6 have been associated with increased pruritic severity,^7^^,^^8^ but no mechanistic evidence of IL-6 driving GCA-related pruritus exists. Due to its contribution to various proinflammatory processes, we hypothesize that the overall systemic inflammatory suppression of IL-6 by tocilizumab may have indirectly suppressed processes potentially causing our patient’s pruritus. Notably, the oral corticosteroid therapy is likely responsible for the patient’s initial pruritic relief, due to its administration prior to tocilizumab and the rapid symptom resolution. However, due to its temporary course and the patient’s sustained pruritus resolution, tocilizumab is the more probable contributor to the continued outcome (Fig 1). Although we do not assert to be the first to report GCA-related pruritus, we propose the possibility of an underrecognized association.

In addition to the patient’s dermatologic presentation, the ophthalmologic course veered from typical GCA features. Acute-onset vision loss is well-described in GCA and is commonly due to anterior ischemic optic neuropathy.^9^ Our patient did not demonstrate clinical signs or symptoms of ischemic lesions, and his long glaucoma history is what likely contributed to his glared night vision. Furthermore, despite having peripheral vascular disease, a risk factor of GCA-related vision loss,^10^ he did not exhibit this symptom.

In patients with biopsy proven GCA, about half of them have PMR features.^11^ PMR is significant in context of this clinical presentation in that the patient exhibited typical PMR features, unlike his GCA. In cases like this, physicians may rule out GCA if textbook manifestations are absent, and consequent vision loss may ensue due to delay in diagnostic testing.^9^ This observation highlights the importance of considering inflammatory and rheumatologic etiologies in patients with chronic pruritus without an identifiable dermatologic cause.

## Conflicts of interest

None disclosed.
